# Paradoxical effect of smoking following acute myocardial infarction

**DOI:** 10.1186/1532-429X-15-S1-O107

**Published:** 2013-01-30

**Authors:** Giuliana Durighel, Tim Dawes, Clare Ashwin, Stuart A Cook, Declan P O'Regan

**Affiliations:** 1 Robert Steiner MR Unit, Imperial College, London, UK; 2 Institute of Clinical Science, Imperial College, London, UK

## Background

Cigarette smoking causes coronary endothelial dysfunction and is a major risk factor for ischemic heart disease and acute ST-elevation myocardial infarction (STEMI) [1]. Prior studies have found that the mortality rate of smokers after AMI may paradoxically be lower than in non-smokers [2]. Epidemiological studies have failed to find a dose-dependent relationship between cardiovascular risk and the number of cigarettes smoked [3]. We used CMR to analyse myocardial infarct size and salvage % in patients following primary percutaneous coronary intervention (PPCI), categorising smokers as never-, ex- and current smokers.

## Methods

**Figure 1:**
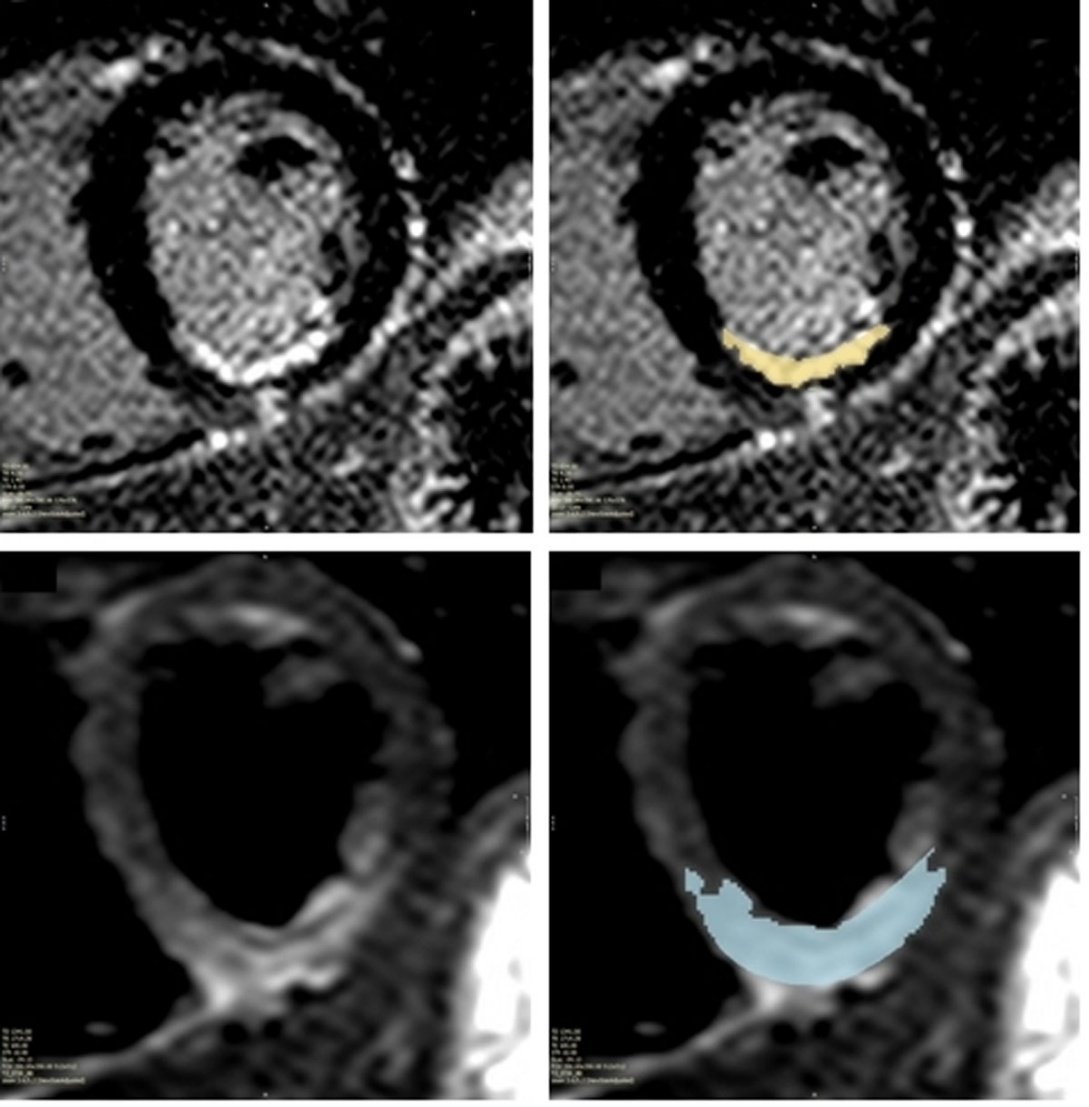
Quantification of myocardial necrosis (upper) and salvage (lower) on MRI following PPCI in a current smoker.

In a prospective single-centre study sixty one patients underwent CMR on a 1.5T Philips Achieva (Best, Netherlands) during the first week following PPCI for acute STEMI. The ischemic area-at-risk was assessed with T2-weighted imaging and myocardial necrosis with late gadolinium enhancement. Myocardial salvage quantification was performed using certified analysis software (cmr42, Circle Cardiovascular Imaging, Alberta, Canada) (Fig 1). Statistical analysis was carried out using SPSS 19.0 (IBM, Armonk, NY).

## Results

**Figure 2:**
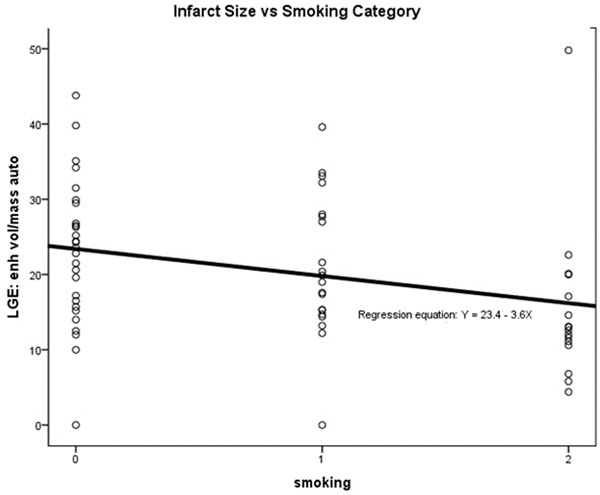
Association of smoking on infarct size. 0= Never Smoked 1= Ex-smoker 2= Current Smoker

Infarct size was normally distributed (by Normal plots and Shapiro Wilk testing). Univariate regression analysis showed smoking status was the only factor significantly associated with infarct size (R2=0.085, p=0.02) (Fig 2). Univariate regression analysis also showed pain-to-balloon time to be the only factor significantly associated with salvage (R2=0.085, p=0.02).

## Conclusions

Smoking is associated with smaller infarcts but is not an independent predictor of myocardial salvage. This study further highlights the debate surrounding the "smokers' paradox". Ischemic pre-conditioning, as well as favourable baseline clinical and angiographic characteristics, may be responsible for the more benign prognosis of current smokers [4]. Adaptations in the coronary microcirculation and the subsequent response to ischemia-reperfusion injury may underlie the pathophysiological differences seen in smokers [5].

## Funding

No external funding sources.
